# Bumetanide Oral Liquid Formulation for the Treatment of Children and Adolescents with Autism Spectrum Disorder: Design of Two Phase III Studies (SIGN Trials)

**DOI:** 10.1007/s10803-020-04709-8

**Published:** 2020-11-05

**Authors:** Véronique Crutel, Estelle Lambert, Pierre-François Penelaud, Cristina Albarrán Severo, Joaquin Fuentes, Antoine Rosier, Amaia Hervás, Stéphane Marret, Guiomar Oliveira, Mara Parellada, Simon Kyaga, Sylvie Gouttefangeas, Marianne Bertrand, Denis Ravel, Bruno Falissard

**Affiliations:** 1 Neuro Immuno-Inflammation Therapeutic Area, Institut de Recherches Internationales Servier, Suresnes, France; 2 Child & Adolescent Psychiatry Service, Policlínica Gipuzkoa & GAUTENA Autism Society, San Sebastián, Spain; 3 Department of Neonatal Pediatrics, CHU de Rouen and CHU Le Rouvray, Sotteville les Rouen, France; 4 Child and Adolescent Mental Health Service, Hospital Universitari Mútua de Terrassa, and Global Institute of Neurodevelopment Integrated Care (IGAIN), Barcelona, Spain; 5 Department of Neonatal Pediatrics, Intensive Care, and Neuropediatrics, Rouen University Hospital, Rouen, France; 6 INSERM U 1245 team 4 Neovasc, School of Medicine, Normandy University, Rouen, France; 7 Neurodevelopmental and Autism Unit from Child Developmental Center and Centro de Investigação e Formação Clínica, Hospital Pediátrico, Centro Hospitalar e Universitário de Coimbra, Coimbra, Portugal; 8 Faculty of Medicine, University Clinic of Pediatrics, University of Coimbra, Coimbra, Portugal; 9 Servicio de Psiquiatría del Niño y del Adolescente Hospital General Universitario Gregorio Marañón, CIBERSAM, IiSGM, Ibiza 43, Madrid, Spain; 10 Global Medical and Patient Affairs, Servier, 35 rue de Verdun, 92284 Suresnes cedex, Suresnes, France; 11 Neurochlore, Marseille, France; 12 University Paris-Sud, Univ. Paris-Descartes, AP-HP, INSERM U1178, Paris, France

**Keywords:** Autism spectrum disorder, Bumetanide, Pediatrics, Randomized controlled trial

## Abstract

There are currently no approved pharmacological treatments to improve social reciprocity and limit repetitive and rigid behaviors in autism spectrum disorder (ASD). We describe the design of two Phase III studies evaluating the efficacy/safety of bumetanide oral liquid formulation in ASD. These are international, multicenter, randomized, double-blind, placebo-controlled studies in children and adolescents with ASD aged 7 to 17 years (n = 200; study 1), or younger children with ASD aged 2 to 6 years (n = 200; study 2). The primary endpoint of each is change in Childhood Autism Rating Scale 2 total raw score after 6 months. These studies could contribute to the first pharmacological treatment to improve social reciprocity and limit repetitive and rigid behaviors in children and adolescents with ASD.

**Supplementary Information:**

The online version contains supplementary material available at 10.1007/s10803-020-04709-8.

Autism spectrum disorder (ASD) is a lifelong neurodevelopmental condition characterized by an early onset of persistent deficits in communication and social interaction across multiple contexts, and the presence of restrictive, repetitive patterns of behaviors, interests, or activities (American Psychiatric Association 2013, 2016). Today, no pharmacological treatments exist for these symptoms. Associated symptoms and behaviors such as irritability, self-injuriousness, temper tantrums, quickly changing moods, sleep disturbances, anxiety, or depressive disorders are frequently reported (Fitzpatrick et al. 2016; Lai et al. 2019). Between 69 and 79% of individuals with ASD experience at least one additional psychiatric condition during their lifetime (Buck et al. 2014; Lever and Geurts 2016). Furthermore, ASD and co-occurring conditions can impose a significant emotional burden and negatively affect the quality of life (QoL) of both patients and their families (Chiang and Wineman 2014; Özgür et al. 2018; Picardi et al. 2018).

ASD is more common in males than females, with a gender ratio of approximately 3:1 in community studies (Loomes et al. 2017). The gender difference may be caused by under- or mis-diagnosis in females (Loomes et al. 2017); however, biological mechanisms have also been proposed (Baron-Cohen et al. 2011). The global prevalence of ASD in children is estimated to be 7.6 cases per 1000 population (Baxter et al. 2015), although estimates have ranged from 0.1 to 18.9 cases per 1000; this wide variation in reported prevalence may be reflective of differences in diagnosis and study methodology (Elsabbagh et al. 2012). In 2016, 62.2 million people worldwide were estimated to have ASD (GBD 2016 Disease and Injury Incidence and Prevalence Collaborators 2017). The varied severity and presentation of ASD can make diagnosis and treatment challenging. The causes of ASD have not been completely elucidated, however a number of genetic and environmental risk factors (and interplay between them) are believed to be involved (Park et al. 2016). Risk factors include previous ASD diagnosis in an older sibling, genetic mutations, older paternal age at the time of conception, preterm birth, intrauterine inflammation, drug exposure during pregnancy and in the perinatal period, and delivery complications (Autism Genome Project Consortium et al. 2007; Bourgeron 2009; Breuss et al. 2020; Casanova et al. 2009; Gardener et al. 2009; Glasson et al. 2004). A recent study suggested that inherited genetic factors present a significant risk factor for ASD, with an estimated heritability of approximately 80% (Bai et al. 2019).

The aims of the current medical interventions for ASD are to manage behavioral disturbances and co-existing conditions, such as sleep disturbances and anxiety, in order to improve QoL (Medavarapu et al. 2019). Educational and behavioral interventions, such as speech and language therapy, Applied Behavior Analysis (ABA), and occupational therapy, are a key part of ASD treatment (Howlin and Moss 2012; Myers et al. 2007; Volkmar et al. 2014). A number of interventions (including parent-mediated communication therapy and those based on developmental models with early, intensive behavioral intervention) have demonstrated improvements in cognitive and communication abilities (Dawson et al. 2010; Pickles et al. 2016; Tonge et al. 2014). There are currently no pharmacological treatments approved to specifically improve social reciprocity and limit repetitive and rigid behaviors in ASD; instead, drug treatments have targeted disruptive behavioral symptoms, such as irritability, aggression, hyperactivity, and sleep disturbances (Farmer et al. 2013; Hong and Erickson 2019). Aripiprazole and risperidone are approved in the US, but not in the European Union (EU), to treat irritability in children and adolescents with ASD, however they are associated with significant side effects (Fung et al. 2016; Marrus et al. 2014). Haloperidol is approved in the EU for the management of persistent, severe aggression in children aged 6 to 17 years with ASD, when other treatments have failed or are not tolerated (EMA 2017). However, the reported side effects of haloperidol include persistent dyskinesis and extrapyramidal symptoms (FDA 2009b). In 2018, prolonged-release melatonin was approved in the EU to treat insomnia in children and adolescents with ASD (EMA 2018).

Bumetanide acts by inhibiting the Na^+^-K^+^-2Cl^−^ cotransporters NKCC1 and NKCC2; it is indicated for the treatment of edema associated with congestive heart failure and hepatic and renal diseases (including nephrotic syndrome) in adults (FDA 2009a), and has a known safety profile (Brater 2000; Howlin and Moss 2012; Wittner et al. 1991). Its activity is related to the inhibition of the NKCC2 cotransporter, which is expressed exclusively in the kidneys (Russell 2000). The NKCC1 cotransporter is widely distributed throughout the body, with a higher expression in the brain, where its inhibition is hypothesized to underlie the therapeutic efficacy of bumetanide in ASD (Ben-Ari 2017; Blaesse et al. 2009).

An oral liquid formulation of bumetanide is currently being assessed as a potential treatment for children and adolescents with ASD. In the adult nervous system, inhibitory neurotransmitter γ-aminobutyric acid (GABA) signaling is involved in sensory and cognitive function. In fetal life and early postnatal development, GABA exerts excitatory actions that contribute to normal central nervous system development (Represa and Ben-Ari 2005). The regulation of neuronal activity by GABA depends strongly on the levels of intracellular chloride, which are finely modulated by NKCC1 and KCC2 (a K^+^-Cl^−^ transporter) (Deidda et al. 2014). An imbalance between excitatory and inhibitory neuronal activity has been associated with impairments in communication skills, language, and sensory perception, which are areas particularly affected in ASD (Lunden et al. 2019).

In children, adolescents, and adults with ASD, persistence or development of excitatory GABA signaling has been observed, and the characteristic symptomatology of ASD could reflect an imbalance in the ratio of excitatory to inhibitory synaptic transmission (Nelson and Valakh 2015). Moreover, enhanced activity of NKCC1 has been found to increase excitatory GABA signaling effects, intracellular chloride levels, and neuronal depolarization (Ben-Ari 2017; Grandgeorge et al. 2014). Pharmacological studies in animal models of ASD showed the ability of bumetanide to restore the inhibitory action of GABA and to attenuate the behavioral features of autism (Eftekhari et al. 2014; He et al. 2014; Nardou et al. 2011; Tyzio et al. 2014). It is thought that bumetanide may reduce ASD symptoms by promoting inhibitory GABA signaling through normalization of intracellular chloride levels (Bruining et al. 2015).

Previous pilot and Phase II clinical studies of bumetanide oral liquid formulation in children and adolescents with ASD have shown improvement in social reciprocity and adaptive behavior as measured by the Childhood Autism Rating Scale (CARS), Social Responsiveness Scale (SRS), and in visual recognition of emotive figures (Hadjikhani et al. 2015; Lemonnier and Ben-Ari 2010; Lemonnier et al. 2012, 2017). The most recent Phase II study randomized 88 children aged 2 to 18 years with ASD to bumetanide (0.5, 1.0, or 2.0 mg twice daily) or placebo treatment for 3 months. In patients who completed the study, mean CARS score significantly improved with bumetanide compared with placebo, SRS score improved by > 10 points, and mean Clinical Global Impression (CGI)-I score also significantly improved. In bumetanide-treated patients, the most frequently reported adverse events (AEs) included hypokalemia, dehydration, and diuresis-related events. The incidence and severity of AEs increased with dose, however no dose–response effect for efficacy was observed (Lemonnier et al. 2017). The BAMBI (Bumetanide in Autism Medication and Biomarker) trial is a placebo-controlled, randomized, investigator-initiated trial of bumetanide oral liquid formulation in children (n = 92) aged 7 to 15 years with unmedicated ASD. The study assessed whether treatment with bumetanide oral liquid formulation for 3 months improved symptoms of ASD, as measured by the SRS-2 total score. As a secondary endpoint, cognitive and neurophysiological assessments (behavioral questionnaire, neurocognitive test battery, event-related potential paradigms, and resting state paradigms) were used to identify prognostic biomarkers of bumetanide oral liquid formulation treatment, with a view to enabling a more personalized treatment approach in the future. The BAMBI study did not find that bumetanide was superior to placebo based on the primary outcome (SRS-2 total score), although a superior effect was reported based on a secondary endpoint (Repetitive Behavior Scale-Revised; Sprenger et al. 2020). Another recent study of bumetanide in children (n = 83) with ASD aged 3 to 6 years demonstrated significant benefits compared with control, using the CARS as the primary outcome measure (Zhang et al. 2020). An earlier study also investigated whether bumetanide may provide additional improvements in the outcome of children (n = 60) with ASD aged 2.5 to 6.5 years receiving ABA (Du et al. 2015). Findings suggested that bumetanide in combination with ABA may indeed result in a better outcome for children with ASD than ABA alone.

In this report, we describe the design of two Phase III studies evaluating the efficacy and safety of bumetanide oral liquid formulation for the treatment of ASD in children and adolescents.

## Study Design

Both Phase III studies are of the same design; randomized, double-blind, and placebo-controlled. One study includes children and adolescents aged 7 to 17 years and the other includes younger children aged 2 to 6 years. It is expected that each study will include 200 patients from approximately 50 centers across 13 countries.

**Fig. 1:**
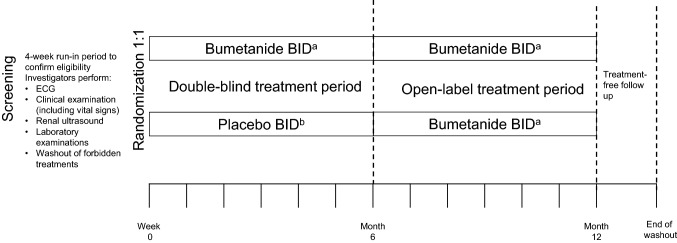
Study design. *BID* twice daily, *ECG* electrocardiogram. ^a^Bumetanide oral liquid formulation; dose adapted by body weight. Patients < 25 kg will receive bumetanide 0.02 mg/kg BID (0.04 ml/kg oral liquid formulation BID); patients ≥ 25 kg will receive bumetanide 0.5 mg BID (1 ml oral liquid formulation BID); ^b^Patients < 25 kg will receive placebo oral solution of 0.04 ml/kg BID; patients ≥ 25 kg will receive placebo oral solution 1 ml BID

The study design is shown in Fig. 1. Following the selection visit, patients enter a run-in period of up to 4 weeks prior to randomization to confirm eligibility. At the inclusion visit (Week 0), eligible patients are randomized (1:1 ratio) to one of two parallel treatment groups, bumetanide oral liquid formulation twice daily (BID) or placebo BID, and enter a 6-month double-blind treatment period (Week 0 to Week 26). Randomization is stratified by country and patient sex.

At Week 26, patients enter a 6-month open-label treatment period (Week 26 to Week 52). Patients randomized to bumetanide oral liquid formulation in the double-blind treatment period continue to receive bumetanide BID. Patients randomized to placebo receive bumetanide BID in the open-label treatment period. Finally, a follow-up period of 6 weeks is completed at the end of the study (earlier in the case of premature discontinuation from the study) to assess efficacy and safety after treatment discontinuation.

In order to maintain the double-blind treatment conditions, CARS2 is assessed by independent raters, who do not have access to patient data and are not involved in any aspect of the patient’s management. All independent raters undergo mandatory training for CARS2 before their involvement in the study.

### Patients

Informed assent/consent of the patient and parents/legal representative(s) or caregivers (where applicable) is collected for every patient at the selection visit, before any study-related procedures are carried out. The patient selection phase occurs before inclusion in the study, based on the following criteria: male or female; aged from 7 to 17 years (for study 1) or 2 to 6 years (for study 2); outpatients (including patients living in an institution); living with their parent/legal representative or caregiver; primary diagnosis of ASD as per DSM-5 criteria (American Psychiatric Association 2013, 2016); ASD criteria met on the Autism Diagnostic Observation Schedule (ADOS-2) and Autism Diagnostic Interview Revised (ADI-R); and moderate to severe ASD according to CGI severity rating score ≥ 4, CARS2 (ST or HF) total raw score ≥ 34, and SRS-2 (pre-school version or school age version) ≥ 66 T-Score. Non-selection criteria included: concomitant participation in another study, or previous participation in a study of another medicinal product for 3 months prior to enrollment; known monogenic syndrome (e.g. Fragile X, Rett Syndrome); high suicide risk or psychiatric conditions considered likely to interfere with the conduct of the study; chronic hepatic disease, renal dysfunction or cardiac dysfunction; unstable psychotherapy, behavioral, cognitive, or cognitive-behavioral therapy; concomitant psychotropic medication (exceptions: aripiprazole and risperidone in study 1, which are permitted if a stable dose is used between selection and inclusion, and up to Week 26; methylphenidate, atomoxetine, or guanfacine, which are permitted in both studies if stabilized for at least 4 weeks prior to inclusion and not planned to be modified or stopped up to Week 26) or other contraindicated medication; and previous treatment with bumetanide that was not effective for the treatment of ASD symptoms.

Patients meeting these requirements will enter the study and complete the 4-week run-in period. At the inclusion visit, a review of patient eligibility is carried out prior to randomization. Inclusion and non-inclusion criteria are listed in Table 1.

**Table 1:** Inclusion and non-inclusion criteria

Inclusion criteria	Non-inclusion criteria
• Patient still fulfilling all the selection criteria • CARS2 (ST or HF) total raw score ≥ 34 • SRS-2 (parent report-school age version) ≥ 66 T-Score • a T-score of 66 corresponds to a total raw score of 73 to 75 for male patients and a total raw score of 66 to 68 for female patients • Patient respecting washout periods for forbidden treatments, considering these treatments were not or poorly effective when applicable	• Any non-selection criterion that could have appeared after the selection visit • Patients having already been treated with bumetanide for ASD (with clinical benefit) but stopped less than 3 months prior to inclusion • Any clinically significant abnormality detected during screening period that is likely to interfere with the study conduct or evaluation: • physical examination, • renal ultrasonography especially unique kidney, renal hypoplasia, renal dysplasia, nephrocalcinosis, renal hyperechogenicity, loss of corticomedullary differentiation (list not exhaustive) • ECG especially long QT interval (QTCF ≥ 460 ms) • laboratory test especially hypokalemia (K < 3.5 mEq/L) and clinically relevant hypercalciuria (in the case of abnormal urinary calcium/creatinine ratio that may be the expression of clinically relevant hypercalciuria, a retest should be performed as soon as possible. Based on these results, the investigator should have a discussion with the local pediatric nephrologist in order to assess whether it corresponds to the presence of a clinically relevant hypercalciuria according to their clinical judgment [based on broader sources, including risk factors, medical history, clinical examination, and other biological parameters]), positive for hepatitis A or hepatitis B serology not explained by a vaccination or by a past resolved infection, positive for hepatitis C serology • Clinically relevant nephropathy according to the clinical investigator’s judgment (based on broader sources, including risk factors, medical history, clinical examination, and other biological parameters) • eGFR ≤ 90 ml/min/1.73m^2^ (estimated glomerular filtration rate, Schwartz formula 2009, or the CKD-EPI formula for patients ≥ 13 years old whose height is ≥ 150 cm and weight ≥ 45 kg) • Severe electrolyte imbalance that is likely to interfere with the study conduct or evaluation Positive urinary drug screening not explained by a known use of an authorized substance (e.g. codeine, methylphenidate) • Positive pregnancy test (βHCG) for all post-pubertal females • Patient who has a current suicide risk according to the investigator (based on the information obtained during the evaluation of the C-SSRS-C [Children version] Baseline/Screening: ‘suicidal ideation’ part, item 4 or 5 is ‘yes’ in ‘6 months’ part)

*ASD* autism spectrum disorder, *βHCG* beta human chorionic gonadotropin, *CARS2* Childhood Autism Rating Scale, Second Edition, *CKD-EPI* Chronic Kidney Disease Epidemiology Collaboration, *C-SSRS-C* Columbia Suicide Severity Rating Scale Children’s version, *ECG* electrocardiogram, *eGFR* estimated glomerular filtration rate, *HF* high-functioning version, *QTCF* corrected QT interval by Fredericia, *SRS-2* Social Responsiveness Scale, *ST* standard version

### Study Treatment

In order to maintain double-blinding of the study, bumetanide oral liquid solution and placebo will have the same appearance. The volume of administered solution will be adapted to patient body weight (0.04 ml/kg for patients < 25 kg). Patients randomized to bumetanide treatment who weigh < 25 kg will receive bumetanide 0.02 mg/kg BID (oral solution 0.04 ml/kg BID); and patients weighing ≥ 25 kg will receive bumetanide 0.5 mg BID (oral solution 1 ml BID). Patients in the placebo group weighing < 25 kg will receive a placebo solution BID (with volume adapted to their weight); patients ≥ 25 kg will receive a placebo solution of 1 ml BID.

### Objectives and Assessments

The study objectives and associated endpoints are described in Table 2. The primary objective is to demonstrate superiority of bumetanide compared with placebo after 6 months of treatment, which will be evaluated in terms of change from Week 0 to Week 26 in CARS2. The secondary objectives are to assess the effect of bumetanide oral liquid formulation on other efficacy endpoints, assess the safety of bumetanide, confirm the acceptability and tolerability of the oral liquid formulation, describe the effects of bumetanide on patient QoL, and to improve the existing pharmacokinetic (PK) model of bumetanide in this population. The effect of bumetanide on utility index scores is an exploratory endpoint.

**Table 2:** Study objectives and endpoints

	Objective	Endpoint(s)
Primary	To demonstrate the superiority of bumetanide 0.5 mg BID oral liquid formulation compared with placebo in the improvement of ASD core symptoms after 6 months of treatment	CARS2 total raw score Main expression will be change from baseline to 6 months
Secondary	To assess the effect of bumetanide on the other efficacy endpoints	• The change in SRS-2 total raw score from baseline to 6 months • CGI-I score at 6 months • The change in VABS II subscores from baseline to 6 months • The change in each individual CARS2 domain from baseline to 6 months
	To assess the safety of bumetanide	• AE, PAERS • Clinical laboratory evaluation • Vital signs and clinical examination: weight (kg), height (m), BMI (kg/m^2^), systolic blood pressure (mmHg), standing, sitting; diastolic blood pressure (mmHg), standing, sitting, heart rate (bpm) • Electrocardiogram • Renal ultrasound • Assessment of suicidal ideation and suicidal behavior using the C-SSRS-C • Assessment of pubertal development through Tanner stages (for patients aged 7 to 17 years only)
	To confirm the acceptability and palatability of the oral liquid formulation	• Acceptability and palatability questionnaire
	To describe the effects of bumetanide on patients’ quality of life	• PedsQL expressed in terms of change from baseline to Week 26 • WHOQOL-Brief questionnaire summarized at each planned visit for each period using descriptive statistics
	To improve existing pharmacokinetic model of bumetanide in this population	• Pharmacokinetic points at Week 12 and Week 26
Exploratory	To describe the effect of bumetanide on utility index scores	• Utility index score on the EQ-5D-3L

*AE* adverse event, *ASD* autism spectrum disorder, *BID* twice daily, *CARS2* Childhood Autism Rating Scale, Second Edition, *CGI-I* Clinical Global Impression Scale, *C-SSRS-C* Columbia Suicide Severity Rating Scale Children’s version, *EQ-5D-3L* EuroQol five-dimension three-level questionnaire, *PAERS* Pediatric Adverse Event Rating Scale, *SRS-2* Social Responsiveness Scale, *PedsQL* Pediatric Quality of Life Inventory, *VABS* Vineland Adaptive Behavior Scale, *WHOQOL* World Health Organization Quality of Life

Study data will be collected during scheduled visits in each treatment period and at the follow-up visit. The schedule of study assessments is shown in Table 3.

**Table 3:** Investigation schedule

	Selection (Screening)	Inclusion (Baseline)	Double-blind 26-week treatment period									Visits to complete in case of IMP discontinuation during the double-blind period BUT not withdrawal from the study	
	ASSE	W000	W000 + 48/72 h	W000 + Day 10	W000 + Day 17	W004	W008	W012	W016	W020	W026	W012	W026
Informed consents/assents	X^1^												
Demography	X												
IQ test^2^	X												
DSM-5	X												
ADOS-2^3^	X												
ADI-R^4^	X												
Selection / Non-selection criteria	X												
Inclusion / Non-inclusion criteria		X											
Autism diagnostic history	X												
Medical / surgical history	X												
Previous treatments^5^	X												
Concomitant treatments^5^	X	X	X	X	X	X	X	X	X	X	X	X	X
**IRS**													
Patient number	X												
Randomization		X											
IMP allocation		X				X	X	X	X	X	X		
IMP dispensation		X				X	X	X	X	X	X		
Oral solution volume adaptation to patient weight if needed		X						X			X		
Compliance IMP						X	X	X	X	X	X		

	Selection	Inclusion	Double-blind 26-week treatment period									Visits to complete in case of IMP discontinuation during the double-blind period BUT not withdrawal from the study	
	*ASSE*	W000	W000 + 48/72 h	W000 + Day 10	W000 + Day 17 (D017)	W004	W008	W012	W016	W020	W026	W012	W026
**Efficacy measurements**													
CARS2-HF*/* CARS2-ST/ CARS2-QPC		X				X		X			X	X	X
CGI-S	X	X						X			X	X	X
CGI-I					X	X	X		X	X			
SRS-2		X				X		X			X	X	X
VABS II		X									X		
**Safety measurements**													
Suicidality (C-SSRS-C)		X						X			X		
Adverse events		X	X	X	X	X	X	X	X	X	X	X	X
PAERS scale		X			X	X	X	X	X	X	X		
Laboratory tests (blood and urine)	X							X			X		
Blood electrolytes monitoring (K, Na)	X		X	X	X	X	X^(6)^	X	X^(6)^	X^(6)^	X		
ECG	X^(7)^					X^(8)^	X^(8)^	X^(8)^			X^(8)^		
Renal ultrasound	X										X^(8)^		
Sitting and standing blood pressure/heart rate	X	X			X			X			X		
Body weight and height	X	X^(9)^			X^(9)^	X^(9)^	X^(9)^	X	X^(9)^	X^(9)^	X		
Tanner Stage^(10)^		X									X		
**Other measurements for inclusion**													
Urinary drug screening	X												
βHCG—Blood Pregnancy test^(11)^	X												
**Pharmacokinetics (blood samples)**								X			X		
**Other measurements**													
PedsQL		X				X		X			X		
WHOQOL-brief		X				X	X	X			X		
EQ-5D-3L		X						X			X		
Acceptability questionnaire											X		

	Open-label 26-week active treatment period									FU	WD Withdrawal visit to be undergone in case of IMP definitive discontinuation or in case of total withdrawal from the study, whatever the period		
	W026 + 48/72 h	W026 + Day 10	W026 + Day 17 (D199)	W030	W034	W038	W042	W046	W052	Wend			
Concomitant treatments^(5)^	X	X	X	X	X	X	X	X	X	X	X		
**IRS**													
IMP allocation				X	X	X	X	X					
IMP definitive discontinuation											X		
IMP dispensation				X	X	X	X	X					
Oral solution volume adaptation to patient weight						X							
Compliance IMP				X	X	X	X	X	X		X		
**Efficacy measurements**													
CARS2-HF*/* CARS2-ST/CARS2-QPC						X			X	X	X^(12)^		
CGI						X^(13)^			X	X	X		
SRS-2						X			X		X^(12)^		
VABS II									X		X^(12)^		
**Safety measurements**													
Suicidality (C-SSRS-C)						X			X		X		
Adverse events	X	X	X	X	X	X	X	X	X	X	X		
PAERS scale			X	X	X	X	X	X	X		X		
Laboratory tests (blood and urine)						X			X		X		
Blood electrolytes monitoring (K, Na)	X	X	X	X	X^(6)^	X	X^(6)^	X^(6)^	X		X		
ECG				X^(8)^	X^(8)^	X^(8)^			X^(8)^		X		
Renal ultrasound									X^(8)^		X		
Sitting and standing blood pressure/heart rate			X			X			X		X		
Body weight and height			X^(9)^	X^(9)^	X^(9)^	X	X^(9)^	X^(9)^	X		X		
Tanner Stage^(10)^									X		X		
**Other measurements**													
PedsQL				X		X			X		X^(12)^		
WHOQOL-brief				X	X	X			X		X^(12)^		
EQ-5D-3L						X			X		X^(12)^		
Acceptability questionnaire											X		

*ADI-R* Autism Diagnostic Interview Revised, *ADOS-2* Autism Diagnostic Observation Schedule-Generic, *CARS2-HF* Childhood Autism Rating Scale, Second Edition-high-functioning version, *CARS2-QPC* Childhood Autism Rating Scale, Second Edition-Questionnaire for Parents or Caregivers, *CARS2-ST* Childhood Autism Rating Scale, Second Edition-standard version, *CGI* Clinical Global Impression, *CGI-I* Clinical Global Impression-Improvement Scale, *CGI-S* Clinical Global Impression-Severity Scale, *C-SSRS-C* Columbia Suicide Severity Rating Scale Children’s version, *D* day, *DSM-5* Diagnostic and Statistical Manual of Mental Disorders, 5^th^ Edition, *ECG* electrocardiogram, *EQ-5D-3L* EuroQol five-dimension three-level questionnaire, *FU* follow-up, *IMP* investigational medicinal product, *IRS* Interactive response system*, PAERS* Pediatric Adverse Event Rating Scale, *PedsQL* Pediatric Quality of Life Inventory, *SRS-2* Social Responsiveness Scale Second Edition, *VABS II* Vineland Adaptive Behavior Scale Second Edition, *W* week, *WD* withdrawal, *WHOQOL* World Health Organization Quality of Life

^1^To obtain at the latest at ASSE but before any procedure related to the study

^2^Only to be performed if retrospective exam is not available in the 12 months prior to ASSE

^3^Should not be performed if an ADOS-2 evaluation has been done within the 12 months prior to ASSE and is documented in the site

^4^Only to be performed if retrospective ADI-R is not available after the 4 y.o. of the patient

^5^Including non-pharmacological therapies (psychotherapy, social skills training, behavioral interventions, etc.)

^6^Prescribed based on the clinical opinion of nephrologist or investigator

^7^Triplicate ECG only at ASSE

^8^Results of the exam should be available at this visit, prescription to be done at the previous visit

^9^Only body weight

^10^For patients aged 7 to 17 years only

^11^Only for post-pubertal females

^12^Only to be undergone after the last IMP intake by patients discontinuing prematurely from the study after W000 + Day 10

^13^CGI-I only

### Analysis

For each study, the sample size was estimated to meet the primary endpoint (change in CARS2 from Week 0 to Week 26) using a two-sided Student’s test. With a total of 170 patients per study, statistical significance should be established with a power of 90% and a type-one error of 0.05 for an effect size of 0.5 between the two treatment groups. Assuming a premature withdrawal rate of approximately 15%, the total number of patients to be included is 200 in each study.

Two sets of statistical analyses will be performed. The main analysis will be carried out at the end of the double-blind treatment period and the second analysis at the end of the open-label treatment period. All efficacy analyses will be conducted on the randomized set (RS, equivalent to intent-to-treat), which will include all patients who are randomized to a treatment arm using the Interactive Response System at Week 0. All safety analyses will be conducted on the safety set (SS), which will include all patients having received at least one dose of study drug.

The primary estimand is defined according to the primary objective of the trial, which is to evaluate the treatment effect, taking into account treatment discontinuation due to AEs or lack of efficacy and independently of treatment discontinuation for non-medical reasons since those patients would have theoretically continued to be treated as planned.

For the analysis of the primary endpoint, bumetanide will be compared with placebo using a general linear model with baseline CARS2 and stratification factors as covariates. In patients who discontinue treatment due to an AE or lack of efficacy, CARS2 values after treatment discontinuation will be considered as missing and imputed using a reference-based multiple imputation with a jump-to-reference approach. CARS2 values after treatment discontinuation due to other reasons will be considered as missing and imputed using a multiple imputation approach. Sensitivity analyses will be performed to assess robustness of the method for handling missing data. Supplementary analyses on the primary endpoint will be performed using a mixed model for repeated measurements. The primary analysis will also be repeated for the CARS2 total score expressed in terms of change from Week 0 to Week 12. The CARS2 total score and subscores will be summarized by treatment group using descriptive statistics.

In the analysis of secondary efficacy endpoints, SRS total score will be analyzed in a similar approach to CARS. CGI-I at Week 26 will be analyzed as a categorical variable and also as a dichotomized responders variable. For the first analytical approach, a Robust General Linear Model using a rank-based analysis (Wilcoxon scores) will be used. For the second, a chi-square test will be applied. Vineland II Adaptative Behavior Composite score will be analyzed using the same approach as for the primary endpoint. QoL (measured with PedsQL and WHOQOL) and index utility (derived from EQ-5D-3L) will be summarized by treatment group using descriptive statistics.

Safety analyses will include incidence and severity of AEs. Results will be summarized descriptively. To assess the PK profiles, a population model will be built using bumetanide concentration–time data collected in the Phase III studies as well as data from a Phase IIb study. The potential influence of covariates will be also investigated. Secondary PK parameters will be derived from the model for each patient, including area under the concentration–time curve at Week 12 (AUC_12_), plasma concentration at steady state (C_ss_), and minimum observed serum concentration (C_min_).

## Discussion

At present, there are no approved drug therapies to improve social reciprocity and limit repetitive and rigid behaviors in ASD. Aripiprazole and risperidone are approved in the US to treat behavioral symptoms; however, consensus guidelines do not recommend their routine use due to the risk of side effects (Howes et al. 2018). Based on preclinical findings, as well as promising preliminary pilot studies and Phase II data, bumetanide oral liquid formulation offers the potential to improve social reciprocity and limit repetitive and rigid behaviors in children and adolescents with ASD. The two Phase III clinical trials described here aim to show superiority of bumetanide versus placebo in these populations.

Two Phase II studies of bumetanide in ASD have been conducted previously; one was a single-center Phase IIa study in 60 children aged 3–11 years (Lemmonier et al. 2012), the other a multi-center Phase IIb study in 88 children and adolescents aged 2–18 years (Lemonnier et al. 2017). In both studies, bumetanide 0.5 mg BID resulted in statistically significant improvements in the CARS over 90 days as compared with placebo, with a similar degree of improvement noted in the bumetanide arm (five points). The Phase IIa study also included a 30-day discontinuation period, after which a worsening of the condition was detected in the bumetanide group (three-point increase in CARS); this provides additional, indirect, evidence for the efficacy of bumetanide in ASD.

The Phase III studies are designed to confirm and extend the findings from the Phase II studies to cover a longer treatment period; a 6-month double-blind treatment period will establish the efficacy and safety of bumetanide, and is followed by a 6-month open-label period that will allow patients initially randomized to the placebo group to receive active treatment. This open-label period will also provide efficacy and safety data over a 12-month period for those initially randomized to bumetanide, allowing assessment of whether efficacy is maintained or even further improved in this group. A 6-week treatment discontinuation period has also been included in the Phase III studies, based on the observed worsening of ASD following discontinuation of bumetanide in the Phase IIa study. The collection of additional data on the course of disease following treatment discontinuation will inform the optimal treatment modalities, in particular the benefits of chronic treatment. Finally, conducting two Phase III studies should provide more robust data on the efficacy of bumetanide in ASD, and a better understanding of the age from which treatment is beneficial (as compared with a single study covering the entire pediatric age range).

The two Phase III studies have a number of strengths. The primary endpoint in the studies (CARS2 at Week 26) is based on data collected during the 6-month double-blind treatment period. All CARS2 assessments in the studies will be performed by an independent assessor to ensure that blinding is maintained. As far as possible, the same assessor will assess CARS2 for a given patient throughout the study as well as all patients at a given site. Additionally, the assessed efficacy endpoints are clinically validated for evaluating symptoms in ASD; CARS2 assesses both social communication and adaptive behavior and is recommended for inclusion in clinical trials by the European Medicines Agency (EMA and CHMP 2017). In the Phase IIa study, CARS was able to capture both improvement with bumetanide treatment and also loss of improvement when treatment was stopped. The improvement in CARS2 total score was recently shown to be strongly correlated with the CGI-I scale (Falissard et al. 2019), which is a well-established tool for measuring clinical improvement in mental disorders. CARS2 can therefore be considered a valid tool for assessing clinical changes of ASD patients in both clinical trials and routine clinical care (Falissard et al. 2019). An additional benefit to the use of CARS2 as the primary endpoint is that it is completed by an independent assessor rather than by parents/guardians (as for scales such as SRS). Also, it was used in the previous clinical studies of bumetanide in ASD (Lemonnier et al. 2012, 2017), and will therefore facilitate pooling of data for meta-analysis. Another strength of the studies is the broad patient inclusion criteria; patients aged 2 to 17 years will be included, as well as patients with an intellectual quotient of < 70 (hence patients with intellectual disabilities can be enrolled in the trials).

Patient selection criteria include a primary diagnosis of ASD as per DSM-5 criteria, plus ASD criteria met on ADOS-2 and ADI-R. Diagnosis based on DSM-5 criteria is rarely used in clinical practice, however ADOS and ADI-R are widely used and recommended in the EMA guidelines on the clinical development of medicines for the treatment of ASD (EMA and CHMP 2017). ADOS and ADI-R were requested to be included by the EMA during discussion of the pediatric clinical development plan for bumetanide in ASD so that diagnosis can be clearly established.

In Phase II studies of bumetanide oral liquid formulation in children and adolescents with ASD, the most commonly reported AEs were related to diuresis and dehydration (Lemonnier et al. 2012, 2017). Hypokalemia linked to the effects of bumetanide can be managed with diet/potassium supplementation, hence these side effects are unlikely to limit the widespread use of bumetanide (at appropriate doses) in children/adolescents with ASD. Increased diuresis associated with bumetanide treatment could potentially jeopardize the blinding in the Phase III studies reported here. However, this effect was found to be limited in both the BAMBI study and the Phase II trial, as the psychiatrist who assessed outcome measures was blinded to the treatment (Lemonnier et al. 2017; Sprengers et al. 2020). Similarly, the primary endpoint in the Phase III studies is being assessed by an independent rater.

### Summary

This Phase III program will provide further data on the long-term efficacy and safety of bumetanide oral liquid formulation in children and adolescents with moderate-to-severe ASD. If positive, the outcome of these studies could contribute to the first pharmacological treatment to improve social reciprocity and limit repetitive and rigid behaviors in ASD, thereby promoting adaptive behavior and improving QoL for patients with ASD, and their families.

## Supplementary Information

Below is the link to the electronic supplementary material.

Supplementary material 1 (MP4 35289 kb)

Supplementary material 2 (PDF 222 kb)
