# Correction to: Bumetanide Oral Liquid Formulation for the Treatment of Children and Adolescents with Autism Spectrum Disorder: Design of Two Phase III Studies (SIGN Trials)

**DOI:** 10.1007/s10803-020-04822-8

**Published:** 2021-01-09

**Authors:** Véronique Crutel, Estelle Lambert, Pierre-François Penelaud, Cristina Albarrán Severo, Joaquin Fuentes, Antoine Rosier, Amaia Hervás, Stéphane Marret, Guiomar Oliveira, Mara Parellada, Simon Kyaga, Sylvie Gouttefangeas, Marianne Bertrand, Denis Ravel, Bruno Falissard

**Affiliations:** 1 Neuro Immuno-Inflammation Therapeutic Area, Institut de Recherches Internationales Servier, Suresnes, France; 2 Child & Adolescent Psychiatry Service, Policlínica Gipuzkoa & GAUTENA Autism Society, San Sebastián, Spain; 3 Department of Neonatal Pediatrics, CHU de Rouen and CHU Le Rouvray, Sotteville les Rouen, France; 4 Child and Adolescent Mental Health Service, Hospital Universitari Mútua de Terrassa, and Global Institute of Neurodevelopment Integrated Care (IGAIN), Barcelona, Spain; 5 Department of Neonatal Pediatrics, Intensive Care, and Neuropediatrics, Rouen University Hospital, Rouen, France; 6 INSERM U 1245 team 4 Neovasc, School of Medicine, Normandy University, Rouen, France; 7 Neurodevelopmental and Autism Unit from Child Developmental Center and Centro de Investigação e Formação Clínica, Hospital Pediátrico, Centro Hospitalar e Universitário de Coimbra, Coimbra, Portugal; 8 Faculty of Medicine, University Clinic of Pediatrics, University of Coimbra, Coimbra, Portugal; 9 Servicio de Psiquiatría del Niño y del Adolescente Hospital General Universitario Gregorio Marañón, CIBERSAM, IiSGM, Ibiza 43, Madrid, Spain; 10 Global Medical and Patient Affairs, Servier, 35 rue de Verdun, 92284 Suresnes cedex, Suresnes, France; 11 Neurochlore, Marseille, France; 12 University Paris-Sud, Univ. Paris-Descartes, AP-HP, INSERM U1178, Paris, France

## Correction to: Journal of Autism and Developmental Disorders 10.1007/s10803-020-04709-8

The author of the article would like to add a video abstract as a supplementary material for a published article. The supplementary file is published with this correction.

The original article has been corrected.

## Supplementary Information

Below is the link to the electronic supplementary material.

Supplementary material 1 (MP4 35289 kb)

