# Supplementary material for: Bumetanide Oral Liquid Formulation for the Treatment of Children and Adolescents with Autism Spectrum Disorder: Design of Two Phase III Studies (SIGN Trials)
Source: J Autism Dev Disord. 2020 Nov 5;51(8):2959–72. doi: 10.1007/s10803-020-04709-8 (PMC8254707; doi:10.1007/s10803-020-04709-8)
Supplement: Supplementary file 2 — Supplementary material 2 (PDF 222 kb) [file 10803_2020_4709_MOESM2_ESM.pdf]

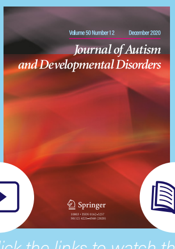

Click the links to watch the video and find out more.

Bumetanide Oral Liquid Formulation for the Treatment of Children and Adolescents with Autism Spectrum Disorder: Design of Two Phase III Studies (SIGN Trials)

Véronique Crutel, Estelle Lambert, Pierre-François Penelaud, Cristina Albarrán Severo, Joaquin Fuentes, Antoine Rosier, Amaia Hervás, Stéphane Marret, Guiomar Oliveira, Mara Parellada, Simon Kyaga, Sylvie Gouttefangeas, Marianne Bertrand, Denis Ravel, Bruno Falissard. *Journal of Autism and Developmental Disorders* https://doi.org/10.1007/s10803-020-04709-8

Autism spectrum disorder (ASD)

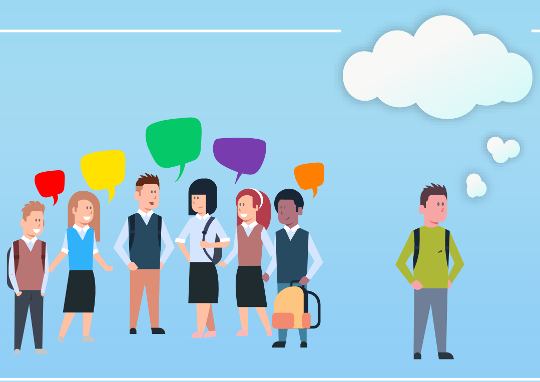

A neurodevelopmental condition with early **deficits in communication** and **social interaction**, and the presence of **restrictive, repetitive behaviors.**

~1% of the population

3:1 male to female ratio

No approved medication for these symptoms

Bumetanide

In ASD, bumetanide is hypothesized to act by inhibiting the **NKCC1 cotransporter** in the brain and promoting inhibitory GABA signaling through normalization of intracellular chloride levels.

An **oral solution** of bumetanide is being assessed as a potential treatment for children and adolescents with ASD with a dedicated formulation.

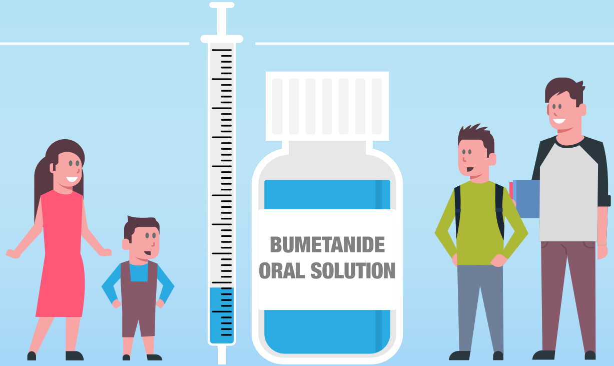

The SIGN trials

13 countries

2 trials

400 total patients

200 autistic children aged 2–6 years

200 autistic children and adolescents aged 7–17 years

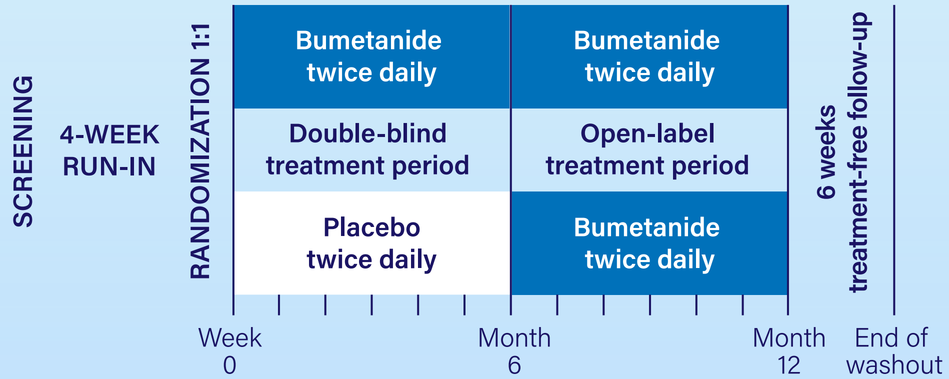

Patients <25 kg: oral solution of bumetanide 0.02 mg/kg twice daily, or placebo
Patients ≥25 kg: oral solution of bumetanide 0.5 mg twice daily, or placebo

Primary endpoint: CARS-2* EMA

Secondary Endpoints: Efficacy, Safety, QoL

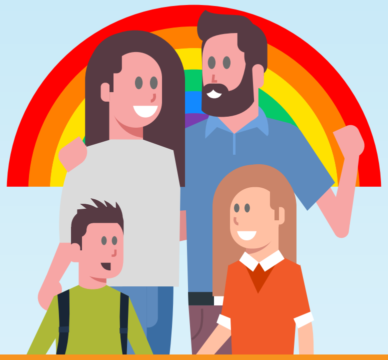

Conclusion

Positive results would herald bumetanide oral solution as the first and only pharmacological treatment to **enhance social reciprocity and limit repetitive behavior**, thereby helping patients and families living with ASD to improve their **social integration and quality of life.**

*The Childhood Autism Rating Scale – Second Edition. EMA, European Medicines Agency; QoL, quality of life.
